# Credibility Analysis of Putative Disease-Causing Genes Using Bioinformatics

**DOI:** 10.1371/journal.pone.0064899

**Published:** 2013-06-05

**Authors:** Olubunmi Abel, John F. Powell, Peter M. Andersen, Ammar Al-Chalabi

**Affiliations:** 1 King's Health Partners Centre for Neurodegeneration Research, King's College London, Department of Clinical Neuroscience, London, United Kingdom; 2 Department of Neuroscience, King's College London, London, United Kingdom; 3 Institute of Pharmacology and Clinical Neuroscience, Section for Neurology, Umeå University, Umeå, Sweden; 4 Department of Neurology, University of Ulm, Ulm, Germany; Pasteur Institute of Lille, France

## Abstract

**Background:**

Genetic studies are challenging in many complex diseases, particularly those with limited diagnostic certainty, low prevalence or of old age. The result is that genes may be reported as disease-causing with varying levels of evidence, and in some cases, the data may be so limited as to be indistinguishable from chance findings. When there are large numbers of such genes, an objective method for ranking the evidence is useful. Using the neurodegenerative and complex disease amyotrophic lateral sclerosis (ALS) as a model, and the disease-specific database ALSoD, the objective is to develop a method using publicly available data to generate a credibility score for putative disease-causing genes.

**Methods:**

Genes with at least one publication suggesting involvement in adult onset familial ALS were collated following an exhaustive literature search. SQL was used to generate a score by extracting information from the publications and combined with a pathogenicity analysis using bioinformatics tools. The resulting score allowed us to rank genes in order of credibility. To validate the method, we compared the objective ranking with a rank generated by ALS genetics experts. Spearman's Rho was used to compare rankings generated by the different methods.

**Results:**

The automated method ranked ALS genes in the following order: *SOD1*, *TARDBP*, *FUS*, *ANG*, *SPG11*, *NEFH*, *OPTN*, *ALS2*, *SETX*, *FIG4*, *VAPB*, *DCTN1*, *TAF15*, *VCP*, *DAO*. This compared very well to the ranking of ALS genetics experts, with Spearman's Rho of 0.69 (P = 0.009).

**Conclusion:**

We have presented an automated method for scoring the level of evidence for a gene being disease-causing. In developing the method we have used the model disease ALS, but it could equally be applied to any disease in which there is genotypic uncertainty.

## Introduction

Genetic studies are challenging in many complex diseases, particularly those with limited diagnostic certainty, low incidence and prevalence, or those of old age. Association studies suffer a reduction in power when there is phenotypic heterogeneity resulting from difficulty with diagnosis, and linkage studies are limited because the older generations are not available and the younger generations have not yet reached the age of risk. The result is that genes are reported as causative with varying levels of evidence and it can be difficult for those not in the field to assess how credible any genetic evidence is.

One such condition is amyotrophic lateral sclerosis (ALS). This is an adult onset neurodegenerative syndrome of upper and lower motor neuron degeneration, with a mean age of onset of 56 in diagnosed familial cases (FALS) and 60 to 70 years in apparently sporadic cases, and an average survival of 3 to 5 years from symptom onset [1] [2]. Illustrating the complexity and difficulty in performing genetic research on ALS, the reported frequency of familial ALS varies from 0.8% [3] to 17–18% [4] although all studies agree that most cases are apparently sporadic [5]. There is, however, a genetic basis both to familial and apparently sporadic ALS [6], [7], [8]. All genes reported mutated in familial ALS have also been found mutated in sporadic ALS. Because of the late age of onset and poor prognosis, suitable families are difficult to collect for linkage, and large populations are difficult to collect for association.

The first gene identified for familial ALS was *SOD1* [9] [10]. Through linkage and association studies of SNPs, microsatellites and copy number variants, as well as through direct sequencing of candidate genes and whole exome sequencing using high throughput methods, over 100 genes have now been implicated in the cause of ALS [11]. The level of supporting evidence for each gene or gene variant varies from small to overwhelming, and is in some cases contradictory. Furthermore, the increasing cooperation between ALS researchers internationally, and the understanding that large datasets are needed, coupled with advances in technology, mean that the rate of detection of putative new ALS genes is rapid and increasing. This leads to two immediate problems: first, it is difficult to keep up with what is an “accepted” ALS gene, and second, there is no simple, objective way to define the list of ALS-causing genes. As a result, researchers may find themselves unable to agree on whether any one gene is an ALS gene or not. The situation is further compounded by the loose definition of ALS, which for genetic purposes has a far wider phenotypic definition than most ALS researchers would accept in a clinical setting [12]. For example, ALS2 includes an infantile, slowly progressive upper motor neuron syndrome that is most similar to hereditary spastic paraparesis, rather than an adult onset mixed upper and lower motor neuron syndrome with a poor prognosis for survival. Similarly, ALS with frontotemporal dementia is regarded as a slightly different entity from ALS even though frontotemporal dementia and ALS are in at least some cases a continuum of disease, and in many cases ALS genes and frontotemporal dementia genes are the same as genes for ALS with frontotemporal dementia.

One solution to this problem is to design some method for objectively scoring the level of evidence supporting a gene or gene variant as disease causing. This would have the advantage that the phenotype could be defined by the user, allowing a loose definition or more stringent definition as required.

The ALSoD database stores data on putative ALS genes using information derived from publications and directly input by researchers. We have therefore explored the possibility of using these data to generate a credibility score for ALS genes with the aim of producing a system that can be generalized to other similar conditions.

## Methods

PRISMA revision [13] with respect to development and reporting of results were taken into consideration. (Checklist S1).

### Data Collection

**Figure 1:**
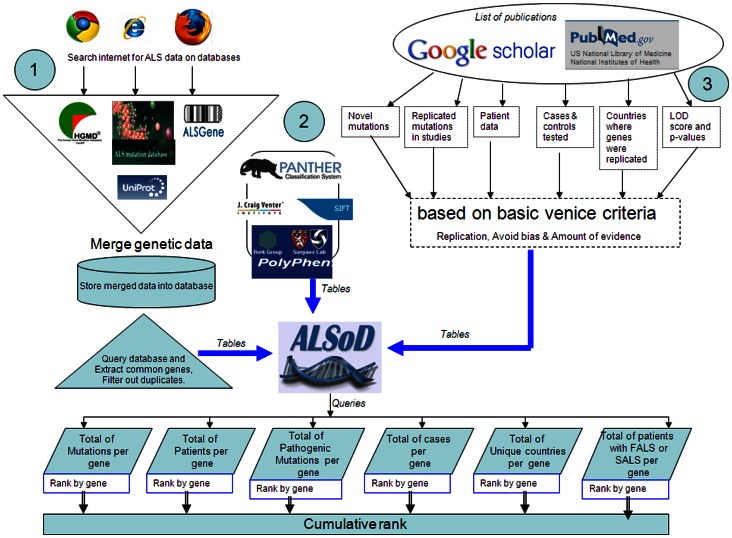
Overview of credibility analysis method.

Genes with at least one publication suggesting involvement in adult onset familial ALS were studied [14]. We excluded genes with limited clinical data, absent mutational data or unreplicated results. Publicly listed variants for the included genes derived from ALSGene, Uniprot, ALS Mutation and HGMD databases were merged with variant lists in ALSoD, and filtered for duplicates (Figure 1).

### Pathogenicity Analysis Using Bioinformatic Tools

PANTHER (Protein Analysis Through Evolutionary Relationships) [15], SIFT (Sorting Intolerant From Tolerant) [16] and POLYPHEN (Polymorphism Phenotyping) [17] programs were used to analyse variants for possible pathogenicity. These tools generated a set of scores for the variants analysed, which for PANTHER are given as a subSPEC (substitution position-specific evolutionary conservation) score and for POLYPHEN given as score differences for PSIC (position-specific independent counts). In PANTHER, all possible mutations for each gene were generated using perl scripts and run on the web service in batches. SubPSEC scores < = −5.0 were defined as damaging and subPSEC scores >−5.0 defined as not damaging. In SIFT, all possible unique codons in each gene were generated using perl script with scores < = 0.05 defined as damaging and scores >0.05 defined as not damaging. In POLYPHEN, all mutations available in a gene on ALSoD were run through the web service one after the other and PSIC score differences > = 1.5 defined as damaging and PSIC score differences <1.5 as not damaging.

### Data Extraction from Publications

We conducted a systematic review of all publications related to ALS genetics with an exhaustive combination of search queries on the 15 genes mentioned above. (Flow diagram S1 and Protocol S1).

In the PubMed database, we used title keywords consisting of the gene name, “mutation” and “ALS” or “Motor Neuron Disease”, or gene name and “novel” to identify key publications and then used the related citations function to generate a list of publications for data extraction. For example, (SOD1[Title] OR (superoxide dismutase[Title]) AND (mutation[Title] OR novel [Title] )AND ((Amyotrophic Lateral Sclerosis[Title]) OR (Motor Neuron Disease[Title]) OR ALS[Title]) yielding 181 results. These results were further filtered by choosing “Humans” as Species and sorted by “Recently Added” thereby displaying 160 unique publications. From the list displayed, we also searched the “Related citations” link on the first publication [10] of the selected gene SOD1 yielding 204 results.

We used Google Scholar (http://scholar.google.co.uk/) to identify publications for import into the ALSoD database, starting with basic search queries to generate a large number of publications. For example, “SOD1” gave about 28,600 results but “SOD1 novel mutations variants ALS “amyotrophic lateral sclerosis” “motor neuron disease” gave 2050 results. We went through the first 20 pages containing 20 publications on each page and already sorted by relevance. Publications with animal models or associated with other diseases were excluded from the long list. A manual comparison with already discovered publications from pubmed was conducted and these were excluded from the list.

Manually curated data extracted from all publications included family history, El Escorial category [18], [19] mutations per gene, number of cases and controls used in the studies, mutations in the same codon, number of patients with family history (FALS), number of patients without family history, mutations replicated in other studies, number of countries replicating the mutation and for linkage studies, LOD scores. Several genes implicated in ALS are also implicated in other diseases, including frontotemporal dementia, spinocerebellar ataxia and parkinsonism. To avoid the problem of non-ALS patients being included in the database, we restricted data curation to publications specifying ALS.

### Automated Gene Ranking

Eleven queries stored as procedures were performed on data collated. These were: 1. The total number of affected patients with El Escorial defined ALS having a mutation in each gene [14], [15]. 2. The total number of ALS affected patients used in each study. This measure was used to account for sampling variance and power [20]. 3. The total number of healthy individuals with a mutation reported in each study. 4. The total number of healthy individuals used in each study. 5. The total number of mutations sharing the same codon. 6. The total number of variants detected in ALS patients for each gene. 7. The total number of mutations with positive pathogenic predictions from the use of the three bioinformatics tools described above. 8. The number of patients with a family history defined as at least one other affected member of the family. 9. The number of patients without a family history of ALS. 10. The number of times a particular variation was replicated across different studies. 11. The number of unique populations where affected patients originated.

**Figure 2:**
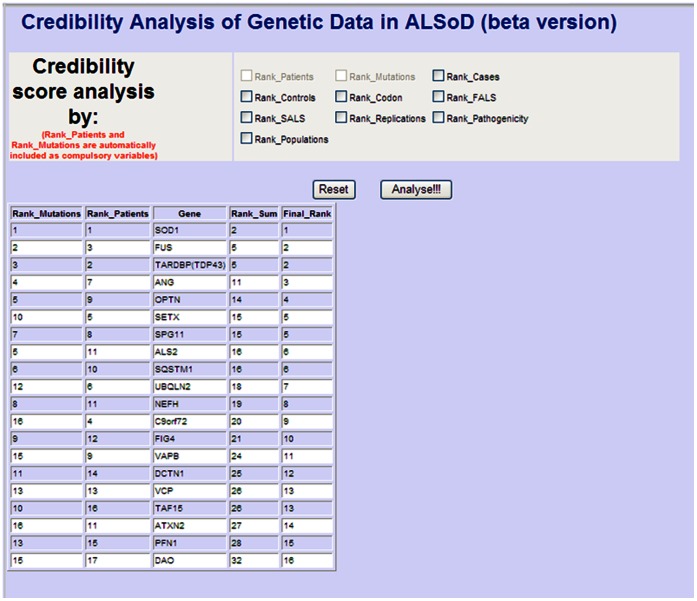
Credibility Analysis webpage.

For each procedure above, a query was generated using Structured Query Language (SQL) on Microsoft SQL Server 2008 and displayed on the ASP.NET platform webpage, ranking the gene. The predicted pathogenicity score for each tool was scored 1 for predicted pathogenic and 0 for predicted not pathogenic and then summed to generate a final score for ranking (http://alsod.iop.kcl.ac.uk/Statistics/pathogenicity.aspx). The rank score for each query was summed to generate an overall rank for the gene under study. For example, from Figure 2, the last row for the *DAO* gene gives the column score 15 for Rank_Mutations, 14 for Rank_Patients and 9 for Rank_Pathogenicity. This produces a total of 38 (that is 15+14+9) in the Rank_Sum column. The generated Rank_Sum for all the genes are arranged in ascending order placing *DAO* 12th by final rank. On the other hand, *FUS* is placed 3rd by final rank as the corresponding scores are 3+3+2 = 8.

There are two possible ways of ranking results in SQL. The default method allocates rank based on the true position, such that if two genes are given equal first position for example, the next gene is in third position, not second. The dense rank method allocates the next gene as second so that there are no gaps in the rank numbering. We used the dense ranking system.

### Validation of the Method

**Figure 3:**
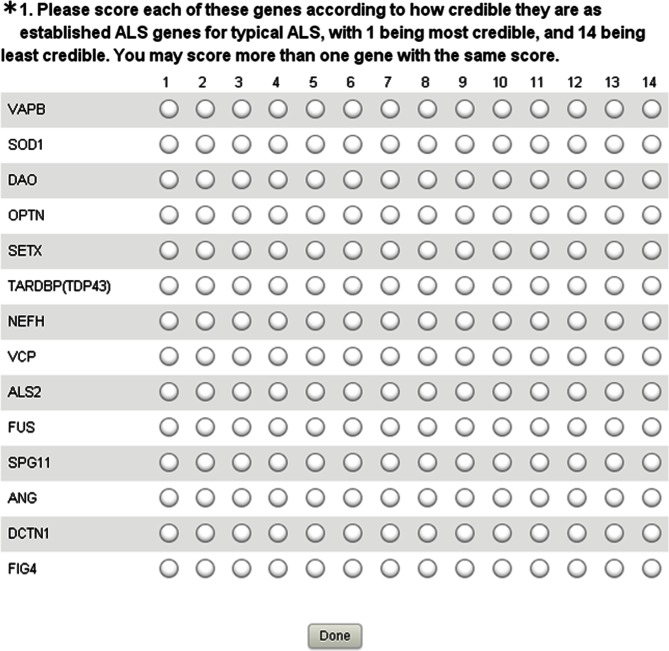
Surveymonkey survey tool for ranking 14 genes.

The purpose of the credibility score tool is to generate a list of genes in order of the weight of evidence supporting involvement in ALS. Such a list should correlate closely with one generated by ALS genetics experts, since such experts should have a good working knowledge of the available evidence. We therefore conducted a survey of ALS genetic experts, defined as being individuals who had published as first or senior author on ALS genetics. Experts were surveyed using the freely available online questionnaire tool, Surveymonkey on http://www.surveymonkey.com/s/WRDW5WT (Figure 3). The survey link showed the genes randomly ordered differently every time the link was clicked to prevent bias in the responses that might occur based on ordering. We also embedded the questionnaire as a submenu on the feedback menu of the ALSoD website. Experts were randomly assigned to one of two groups, one in which the same rank could be assigned to several genes, and one in which responders were forced to rank each gene in order. The first group mimics the final score of the automated method closely, while the second group mimics the detail of the automated ranking method closely, since the automated method is forced to rank each query uniquely but the combined ranking could result in the same value for different genes.

### User Interface

The Credibility Analysis page at (http://alsod.iop.kcl.ac.uk/Statistics/credibility.aspx) allows criteria to be selected by users in the form of checkboxes. Clicking the ‘Analyse’ button then displays the ranked result. A detailed summary of ranked credibility data are also displayed for further reference by users giving the outcome of each procedure based query. Any combination of queries can be included in generating the score except Number of patients and Number of mutations found in each gene which are mandatory selections.

### Statistical Methods

Spearman's Rho [21], [22], [23] was used to compare rankings generated by the automated method and the ALS genetics experts.

## Results

For the pathogenicity prediction, using a threshold score >1 (that is, where the combination score is 2 or 3) to define pathogenicity, just 110 mutations out of 425 were identified as pathogenic, with particularly poor predictions for *FUS* and *TARDBP* when compared with biological evidence of pathogenicity. Using a threshold score of >0 (that is, where the combination score is 1 or 2 or 3) to define pathogenicity brought the number of pathogenic mutations to 198, suggesting that about 50% of recorded FALS mutations are pathogenic based on bioinformatics predictions.

There were 14 genes that fulfilled the inclusion criteria for generation of a credibility score at the time of the survey, and had sufficient data manually curated from publications as explained in the data extraction process above. These were *ALS2*, *FUS*, *DAO*, *VCP*, *VAPB*, *ANG*, *DCTN1*, *FIG4*, *SETX*, *SOD1*, *TARDBP*, *SPG11*, *NEFH*, and *OPTN*.

Using the full set of 11 procedures, the automated method ranked these as ALS-causing genes in the following order: *SOD1*, *TARDBP*, *FUS*, *ANG*, *SPG11*, *NEFH*, *OPTN*, *ALS2*, *SETX*, *FIG4*, *VAPB*, *DCTN1*, *TAF15*, *VCP*, *DAO*.

Subsets of the 11 procedures may be defined by the user if needed. This allows flexibility in which evidence is regarded as useful. For example in Figure 3, using the number of mutations reported in a single gene and the number predicted as pathogenic as test criteria ranks the genes in the following order: *SOD1*, *TARDBP*, *FUS*, *ANG*, *OPTN*, *SETX*, *ALS2*, *SPG11*, *FIG4*, *DCTN1*, *VAPB*, *VCP*, *DAO*. The output shows that the first six genes, *SOD1*, *TARDBP*, *FUS*, *ANG*, *OPTN* and *SETX*, have a total of 121, 17, 19, 12, 5 and 4 pathogenic mutations respectively and, for example, the I113T, D90A and A4V pathogenic mutations of the *SOD1* gene were replicated in 17, 14 and 12 studies. It also shows there are 6 different mutations in codon 93 of *SOD1* and 5 different mutations in codon 521 of *FUS*. Other displayed information includes the number of countries in which gene mutations have been reported. For example, *SOD1* mutation has been reported in 34 countries with representation from every continent of the world, while *TARDBP*, *ALS2*, *ANG*, *FUS*, *SETX* and *NEFH* have been reported in 13, 9, 7, 7, 6 and 5 unique countries respectively. Genes like *FIG4*, *DPP6*, *DCTN1*, *UBQLN2*, *TAF15* which were recorded in only 1 country each have the lowest ranks.

8/25 ALS genetics experts selected based on having published at least one paper on ALS genetics responded. Comparison of the full automated method with the ALS genetics experts' rankings gave a Spearman's Rho of 0.69 (P = 0.009) for the forced expert rankings, and 0.57 (P = 0.042) for the unforced rankings, indicating a good correlation between the methods.

## Discussion

We have presented an automated method for using published information to score the level of evidence supporting a causative relationship between gene mutation and a disease. The information on which the credibility analysis is based is collected routinely by locus-specific databases and the method can therefore be generalized to other diseases. The method used has been applied to amyotrophic lateral sclerosis but could equally be applied to any disease in which there is phenotypic and genotypic heterogeneity.

A strength of this method is that multiple lines of evidence are used to generate an objective opinion as to the credibility of a gene as a disease gene, and while publication bias will affect the score, this is minimized by several factors. First, in this study unpublished data are used since the database includes directly input information from researchers who have not published. Second, a major part of the score is generated using theoretical models of pathogenicity. Third, once published, any information remains useable, and not prone to the vagaries of scientific fashion, or the bias of individual opinion leaders. The effects of these components on the score can be seen by comparing the automated ranking and the ranking generated by both groups of ALS genetics experts. In general the rankings were in agreement. For example, with one exception, the top five genes were the same for all three methods. For some genes there were strikingly different ranks. ANG was ranked 9 of 13 by the experts who could give equal ranks, but in the top five for the other two methods. The biggest discrepancies were otherwise for ALS2, NEFH, and VAPB, each of which was ranked in the bottom two for one of the methods and in the middle for the other two methods.

Similar approaches have been used in association studies. In previous work, three criteria used to determine how credible a disease gene might be were the amount of evidence, manifest as number of studies and population size studied, replicability of a result, and protection from bias by good study design [24]. We have tried to follow similar principles in generating this credibility score.

A weakness of this method is that it relies on an agreed set of criteria for analysis to generate the score, but there is no way to decide objectively whether the criteria are reasonable or what their relative weights should be. For example, we have not included pathogenicity demonstrated in animal models in the score but others might regard this as a vital component. Although we have tried to build in flexibility so that researchers can include or exclude certain criteria, unless the available criteria are exhaustive there will always be the possibility that the method is incomplete. Similarly, because the criteria can be user-selected, there can be no truly universal measure of credibility using this system.

Since this tool was developed, pathological expansion in the *C9orf72* gene has been identified as a cause of ALS and frontotemporal dementia [25], [26]. At the time of our survey of experts this was not the case and it has therefore been excluded from the analysis presented.

A major advantage of this tool is the automation which changes the rank of a gene depending on the evidence provided on the database. This system could be applied to other complex diseases where multiple genes are responsible for a phenotype.

## Supporting Information

Checklist S1 (DOC)

Flow Diagram S1 (DOC)

Protocol S1 (DOC)
