# Supplementary material for: Credibility Analysis of Putative Disease-Causing Genes Using Bioinformatics
Source: PLoS One. 2013 Jun 5;8(6):e64899. doi: 10.1371/journal.pone.0064899 (PMC3674010; doi:10.1371/journal.pone.0064899)
Supplement: Flow Diagram S1 — (DOC) [file pone.0064899.s002.doc]

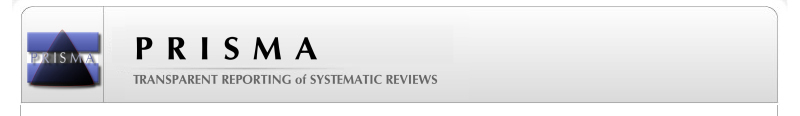
**PRISMA 2009 Flow Diagram**

**Screening**

**Included**

**Eligibility**

**Identification**

Records identified through database searching
(n = 11307)

Additional records identified through other sources
(n = 134)

Records after duplicates removed
(n = 928)

Records screened

(n = 723)

Records excluded

(n = 344)

Articles assessed for eligibility
(n = 379)

Articles excluded

(n = 154)

- *Animal models*
- *Associated with other diseases*
- *same patient cohort*

Studies included in qualitative synthesis
(n = 225)

Studies included in quantitative synthesis (meta-analysis)
(n = 225)
