# Supplementary material for: Credibility Analysis of Putative Disease-Causing Genes Using Bioinformatics
Source: PLoS One. 2013 Jun 5;8(6):e64899. doi: 10.1371/journal.pone.0064899 (PMC3674010; doi:10.1371/journal.pone.0064899)
Supplement: Protocol S1 — (DOC) [file pone.0064899.s003.doc]

**Review protocol**

**Primary database:** PubMed (http://www.ncbi.nlm.nih.gov/pubmed/) and Google Scholar (http://scholar.google.co.uk/)

**Search terms on PubMed:**

1. (SOD1[Title] OR (superoxide dismutase[Title]) AND (mutation[Title] OR novel [Title] )AND ((Amyotrophic Lateral Sclerosis[Title]) OR (Motor Neuron Disease[Title]) OR ALS[Title]) = 181 results
2. ALS2 [Title] OR Alsin[Title] AND (mutation[Title] OR novel[Title]) AND (Amyotrophic Lateral Sclerosis[Title] OR Motor Neuron Disease[Title] OR ALS[Title]) = 6 results
3. ANG [Title] OR Angiogenin[Title] AND (mutation[Title] OR novel[Title]) AND (Amyotrophic Lateral Sclerosis[Title] OR Motor Neuron Disease[Title] OR ALS[Title]) = 6 results
4. (FUS [Title] OR (fusion [Title]) AND (mutation[Title] OR novel [Title] )AND ((Amyotrophic Lateral Sclerosis[Title]) OR (Motor Neuron Disease[Title]) OR ALS[Title]) = 21 results
5. ((TARDBP [Title]) OR (TDP-43 [Title]) OR (fusion [Title])) AND (mutation[Title] OR novel [Title] )AND ((Amyotrophic Lateral Sclerosis[Title]) OR (Motor Neuron Disease[Title]) OR ALS[Title]) = 24 results
6. (VAPB [Title]) OR (Vesicle-associated membrane [Title])) AND (mutation[Title] OR novel [Title] )AND ((Amyotrophic Lateral Sclerosis[Title]) OR (Motor Neuron Disease[Title]) OR ALS[Title]) = 6 results
7. (NEFH [Title]) OR (neurofilament [Title]) AND (mutation[Title] OR novel [Title] )AND ((Amyotrophic Lateral Sclerosis[Title]) OR (Motor Neuron Disease[Title]) OR ALS[Title]) = 1 results
8. (SPG11 [Title] AND (mutation[Title] OR novel [Title] )AND ((Amyotrophic Lateral Sclerosis[Title]) OR (Motor Neuron Disease[Title]) OR ALS[Title]) = 0 result
9. OPTN [Title] OR optineurin [Title] AND (mutation[Title] OR novel[Title]) AND (Amyotrophic Lateral Sclerosis[Title] OR Motor Neuron Disease[Title] OR ALS[Title]) = 11 results
10. SETX [Title] OR Senataxin [Title] AND (mutation[Title] OR novel[Title]) AND (Amyotrophic Lateral Sclerosis[Title] OR Motor Neuron Disease[Title] OR ALS[Title]) = 2 results
11. FIG4 [Title] AND (mutation[Title] OR novel[Title]) AND (Amyotrophic Lateral Sclerosis[Title] OR Motor Neuron Disease[Title] OR ALS[Title]) = 0
12. DCTN1 [Title] OR Dynactin [Title] AND (mutation[Title] OR novel[Title]) AND (Amyotrophic Lateral Sclerosis[Title] OR Motor Neuron Disease[Title] OR ALS[Title]) = 3 results
13. TAF15 [Title] AND (mutation[Title] OR novel[Title]) AND (Amyotrophic Lateral Sclerosis[Title] OR Motor Neuron Disease[Title] OR ALS[Title]) = 1 results
14. (VCP [Title] OR (valosin-containing protein [Title]) AND (mutation[Title] OR novel [Title] )AND ((Amyotrophic Lateral Sclerosis[Title]) OR (Motor Neuron Disease[Title]) OR ALS[Title]) = 4 results
15. (DAO [Title] OR (D-amino-acid oxidase [Title]) AND (mutation[Title] OR novel [Title] )AND ((Amyotrophic Lateral Sclerosis[Title]) OR (Motor Neuron Disease[Title]) OR ALS[Title]) = 1 result

**Search terms on Google Scholar:**

1. SOD1 novel mutations variants ALS "amyotrophic lateral sclerosis" "motor neuron disease" = 2050 results
2. ALS2 novel mutations variants ALS "amyotrophic lateral sclerosis" "motor neuron disease" = 546 results
3. ANG novel mutations variants ALS "amyotrophic lateral sclerosis" "motor neuron disease" = 470 results
4. FUS novel mutations variants ALS "amyotrophic lateral sclerosis" "motor neuron disease" = 2520 results
5. TARDBP TDP-43 novel mutations variants ALS "amyotrophic lateral sclerosis" "motor neuron disease" = 608 results
6. VAPB novel mutations variants ALS "amyotrophic lateral sclerosis" "motor neuron disease" = 396 results
7. NEFH novel mutations variants ALS "amyotrophic lateral sclerosis" "motor neuron disease" = 79 results
8. SPG11 novel mutations variants ALS "amyotrophic lateral sclerosis" "motor neuron disease" = 53 results
9. OPTN novel mutations variants ALS "amyotrophic lateral sclerosis" "motor neuron disease" = 1850 results
10. SETX novel mutations variants ALS "amyotrophic lateral sclerosis" "motor neuron disease" = 1020 results
11. FIG4 novel mutations variants ALS "amyotrophic lateral sclerosis" "motor neuron disease" = 930 results
12. DCTN1 novel mutations variants ALS "amyotrophic lateral sclerosis" "motor neuron disease" = 261 results
13. TAF15 novel mutations variants ALS "amyotrophic lateral sclerosis" "motor neuron disease" = 68 results
14. VCP novel mutations variants ALS "amyotrophic lateral sclerosis" "motor neuron disease" = 404 results
15. DAO novel mutations variants ALS "amyotrophic lateral sclerosis" "motor neuron disease" = 52 results

**Additional databases:**

- The Human Gene Mutation Database at the Institute of Medical Genetics in Cardiff on <http://www.hgmd.cf.ac.uk/ac/index.php>
- ALSGene by the Max Planck Institute for Molecular Genetics Berlin, the Alzheimer Research Forum and Prize4Life on <http://www.alzgene.org/>
- ALS mutation database supported by the Ministry of Education Japan on <http://reseq.biosciencedbc.jp/resequence/SearchDisease.do?targetId=1>
- Online Mendelian Inheritance in Man (OMIM) on <http://www.ncbi.nlm.nih.gov/omim>

**Inclusion criteria:**

1. Amyotrophic Lateral Sclerosis
2. Motor Neuron Disease
3. Mutations/ variants

**Exclusion criteria:**

1. Animal models
2. Associated with other diseases
3. Patients already examined in another study

Study inclusion/exclusion is completed independently (OA & RRG). Results are reviewed (OA) and any disagreement is recorded. Results are discussed (OA & AAC) to reach consensus.

**Data abstraction forms**

**Identification of data abstractor:**

Log in with username and password

1. **Add a mutation**
   1. Select gene
   2. Select mutation location
   3. Select mutation type
   4. Select sequence position and trinucleotide
   5. Select new trinucleotide after mutation
   6. Select zygosity
   7. Select mutation documentation
   8. Input first author
   9. Input year of publication
   10. Input paper title
   11. Input full paper link
   12. Input Doi key (where available)
   13. Input country(s) where mutation is found
   14. Selected Phenotype by default (Amyotrophic Lateral Sclerosis)
   15. Input dbSNP
2. **Submit a patient**
   1. Conduct preliminary screen to check if patient data needs to be submitted to database (like is subject affected?, any mutation found?, any mutations in subject’s family members?)
   2. Select or add a new family id (represented by first author and year e.g Smith 2009)
   3. Select gender
   4. Select country of origin (where not available, consider where research was conducted)
   5. Select ethnic origin (where available)
   6. Select if dead or alive
   7. Choose gene
   8. Has patient been screened?
   9. Was mutation found?
   10. Is there a family history?
   11. Select affected or unaffected status.
   12. Select zygosity
   13. Select mutation
   14. Select site of onset and side of the body
   15. Input age of onset
   16. Input disease duration (in months)
   17. Select UMN or LMN or Cognitive signs
   18. Select phenotype (ALS or FTD or ALS-FTD or Unknown)
3. **Submit a gene**
   1. Input Gene ID  (e.g SOD1)
   2. Input HGNC ID (e.g. 11179)
   3. Input Ensemble ID (e.g. ENSG00000142168)
   4. Input Swissport ID (e.g. P00441)
   5. Input NCBI gene ID (e.g. 6647)
   6. Input NCBI refseq ID (e.g. NM_000454)
   7. Input Structure ID (e.g. uc002ypa.1)
   8. Input OMIM ID (e.g. 147450)
   9. Input Genecards ID (e.g. SOD1)
   10. Input Gene Name (e.g. Cu/Zn superoxide dismutase 1,...)
   11. Input Keywords (e.g. SOD1)
   12. Input Chromosome Name (e.g. 21)
   13. Input Chromosome Position(e.g. q)
   14. Input Chromosome Band (e.g. 22)
   15. Input Chromosome Bp (e.g. 11)
   16. Input Chromosome (e.g. 21q22.11)
   17. Input Protein Name (e.g. superoxide dismutase 1, soluble)
   18. Input Protein Function (e.g.destroys radicals which are.... )
   19. Input Phenotype (e.g. defects in sod1 are the cause of ALS...)
   20. Input Gene comments (e.g. gene: sod1. [153 amino acids; 15 kd])
   21. Input Other_names (e.g. ALS1)
   22. Input Accession ID (e.g. AY04978)
   23. Input reason for investigation (e.g. Mutations in SOD1 account for 20% of familial ALS)
   24. Input Result (e.g. 2-7% sporadic cases have mutations)
   25. Input category (e.g OXIDATIVE STRESS)
   26. Input Gene effect (e.g. FALS genes found in SALS)
   27. Input iHop (e.g. 92317)
   28. Input pdb_id (e.g. 2C9V)
   29. Input dbSNP (e.g. rs92317)
   30. Input pubmed_id
4. **Submit a replicated mutation**
   1. Input Gene ID
   2. Input Mutation
   3. Input Codon
   4. Input Exon
   5. Input Number of Independent families
   6. How many generations were examined?
   7. How many PATIENTS were affected with the mutation? e.g. 5
   8. How many CONTROLS were affected with the mutation? e.g. 0
   9. How many Sporadic ALS patients were examined? e.g. 55
   10. How many Familial ALS patients were examined? e.g 50
   11. Is this mutation reported as SALS or FALS?
   12. Total number of cases examined e.g 15
   13. Total number of controls examined e.g 10
   14. LOD Score reported e.g 6.66
   15. Is a pedigree or Linkage analysis shown?
   16. Input Country of origin
   17. Input Ethnic Origin :
   18. Input SNP (e.g. rs11010):
   19. Is mutation pathogenic ?
   20. Input first Author
   21. Input Year
   22. Input Pubmed ID (e.g. 2020294)

**Characteristics of extracted data:**

<http://alsod.iop.kcl.ac.uk/Overview/gene.aspx?gene_id=SOD1>

<http://alsod.iop.kcl.ac.uk/Overview/gene.aspx?gene_id=ALS2>

<http://alsod.iop.kcl.ac.uk/Overview/gene.aspx?gene_id=ANG>

<http://alsod.iop.kcl.ac.uk/Overview/gene.aspx?gene_id=FUS>

<http://alsod.iop.kcl.ac.uk/Overview/gene.aspx?gene_id=TARDBP(TDP43)>

<http://alsod.iop.kcl.ac.uk/Overview/gene.aspx?gene_id=VAPB>

<http://alsod.iop.kcl.ac.uk/Overview/gene.aspx?gene_id=NEFH>

<http://alsod.iop.kcl.ac.uk/Overview/gene.aspx?gene_id=SPG11>

<http://alsod.iop.kcl.ac.uk/Overview/gene.aspx?gene_id=OPTN>

<http://alsod.iop.kcl.ac.uk/Overview/gene.aspx?gene_id=SETX>

<http://alsod.iop.kcl.ac.uk/Overview/gene.aspx?gene_id=FIG4>

<http://alsod.iop.kcl.ac.uk/Overview/gene.aspx?gene_id=DCTN1>

<http://alsod.iop.kcl.ac.uk/Overview/gene.aspx?gene_id=TAF15>

<http://alsod.iop.kcl.ac.uk/Overview/gene.aspx?gene_id=VCP>

<http://alsod.iop.kcl.ac.uk/Overview/gene.aspx?gene_id=DAO>
